# VEGF Signaling in Neurological Disorders

**DOI:** 10.3390/ijms19010275

**Published:** 2018-01-17

**Authors:** Joon W. Shim, Joseph R. Madsen

**Affiliations:** 1 Department of Medicine, Boston University School of Medicine, Boston, MA 02118, USA; 2 Department of Neurosurgery, Boston Children’s Hospital and Harvard Medical School, Boston, MA 02115, USA; joseph.madsen@childrens.harvard.edu

**Keywords:** vascular endothelial growth factor, cerebrovascular disease, stroke, hydrocephalus, neurological disorders

## Abstract

Vascular endothelial growth factor (VEGF) is a potent growth factor playing diverse roles in vasculogenesis and angiogenesis. In the brain, VEGF mediates angiogenesis, neural migration and neuroprotection. As a permeability factor, excessive VEGF disrupts intracellular barriers, increases leakage of the choroid plexus endothelia, evokes edema, and activates the inflammatory pathway. Recently, we discovered that a heparin binding epidermal growth factor like growth factor (HB-EGF)—a class of EGF receptor (EGFR) family ligands—contributes to the development of hydrocephalus with subarachnoid hemorrhage through activation of VEGF signaling. The objective of this review is to entail a recent update on causes of death due to neurological disorders involving cerebrovascular and age-related neurological conditions and to understand the mechanism by which angiogenesis-dependent pathological events can be treated with VEGF antagonisms. The Global Burden of Disease study indicates that cancer and cardiovascular disease including ischemic and hemorrhagic stroke are two leading causes of death worldwide. The literature suggests that VEGF signaling in ischemic brains highlights the importance of concentration, timing, and alternate route of modulating VEGF signaling pathway. Molecular targets distinguishing two distinct pathways of VEGF signaling may provide novel therapies for the treatment of neurological disorders and for maintaining lower mortality due to these conditions.

## 1. Introduction

Vascular endothelial growth factor (VEGF) is a potent growth factor playing diverse roles in vasculogenesis and angiogenesis. The discovery of VEGF as a permeability factor promoting microvascular permeability in tumors has ignited the research on this polypeptide but its vessel-independent activity has drawn attention recently in the brain [1,2]. One of the fascinating aspects of VEGF among others would be its regulatory role for growth of blood vessels in accordance with outgrowth of nervous network in an orientation opposite to the neural migration in the developing brain. This crosstalk between neurons and vascular niche (microenvironment) is shown to continue after birth and low levels of VEGF contributes to the trophic function or neuroprotection in the central nervous system [3,4].

Bevacizumab—a monoclonal antibody that inhibits all human isoforms of VEGF—is a bestselling oncology drug in a number of types of cancers. Although its cost effectiveness has been argued [5], VEGF inhibition therapy using bevacizumab or a similar class of angiogenesis inhibitors is considered an acceptable approach in cancer. VEGF is potent but a necessary growth factor to be used in maintenance of postnatal vascular and non-vascular systems. Likewise, approaches promoting VEGF and inhibiting VEGF depend on whether VEGF is primary (cause) or secondary (consequence). Pro-VEGF and anti-VEGF therapy alike have been attempted in ischemic stroke. Depending on timing and concentration, the adverse effect was noted in both approaches. Therapeutic use of VEGF has been pursued, showing a rescue as well as edematous effect in ischemic brains and in neurodegenerative disease [2,4,6,7]. VEGF secretion during hemorrhage in utero is pathogenic. Inhibiting downstream pathway of VEGF signaling through angiogenesis inhibitor and/or glucocorticoids has shown a protective effect against germinal matrix hemorrhage [8,9].

Epidermal growth factor receptor (EGFR) in addition to VEGF is a target pathway of solid tumors including breast cancer, which is structurally related with erythroblastic leukemia viral oncogene (*ErbB*) family of proteins. Trastuzumab (Herceptin^®^) is currently used for the treatment of ErbB2 overexpressing breast cancer and HER2/ErbB2 overexpressing metastatic gastric or gastroesophageal junction adenocarcinoma [10,11]. Similar to the cause and effect role for VEGF, ligands of ErbB receptor have shown trophic or pathogenic effect in the non-neoplastic brain. Mice overexpressing one of EGFR/ErbB1 ligands, heparin binding EGF-like growth factor, have been reported to have a hydrocephalic phenotype with subarachnoid hemorrhage (SAH) [12]. Using an early brain injury model, however, it has been recently demonstrated that ErbB4 has a protective effect against neural cell apoptosis in a surgically induced SAH in rats [13].

Therapeutic use of VEGF in a rat model of ischemic stroke through middle cerebral artery occlusion has been well established in the context of neurogenesis, neuroprotection, and angiogenesis starting from infarct core and the penumbra [14]. However, the physiological and pathological contribution of VEGF signaling in cerebrovascular and neurological diseases, especially, in hemorrhagic stroke involving post hemorrhagic hydrocephalus and age-related neurological disease has recently been described [2,12]. The significance of age-related conditions is its progressive development over a relatively longer period of time arising from a transitional state between health and pathological state such as blood pressure rise and vascular stiffening.

The recent Organization for Economic Co-operation and Development (OECD) health statistics data indicated life-modifying aspects about health and mortality. In 2015, the life expectancy of the 35 countries on average was 80.6 years while that of the USA was slightly below the average but kept a pace with other countries with increasing aged populations (78.8 years). The death due to cancer per 100,000 persons of the OECD countries was 203.7 and the USA shows a desired low rate of 187.8 (below the average). The mortality due to cerebrovascular disease such as stroke of the USA (41.5) was much lower than the average of OECD countries (64.5 per 100,000) [15].

The goal of this review is to provide a brief overview of VEGF signaling in cerebrovascular disease such as stroke with SAH, post-stroke hydrocephalus and age-related neurological disorders. Because the leading cause of mortality in the world and in the USA besides cancer is cardiovascular disease, we review status of VEGF and ErbB/EGFR inhibition therapy used in non-neoplastic conditions through a well-established cardiovascular risk factor and consider how the relevant approaches can be translated to the particular neuropathological conditions.

## 2. VEGF Signaling in Cerebrovascular Diseases

The recent review summarized physiological and pathological VEGF in several challenging neurological diseases [2]. We tabulated the point of their description here with an update of hydrocephalus research [9,16]. The most successful VEGF inhibition therapy in non-neoplastic and age-related degeneration is found from ocular disease such as age-related macular degeneration [2]. The role of VEGF is neuroprotective as well as pathological depending on the pathogenic stage and VEGF levels in ischemic and hemorrhagic stroke [17]. We found age- and dose-dependent modification of VEGF function in hydrocephalus. We also found that critical impairment of neuroprotection and vascular activity in amyotrophic lateral sclerosis (ALS), while blood brain barrier (BBB) dysfunction with elevated VEGF is noted in both Alzheimer’s (AD) and Parkinson’s disease (PD). VEGF-mediated BBB dysfunction is commonly described in ALS, PD, and AD [2], but the causal linkage to these age-related neurological conditions is clearer in ALS, in which animal models lacking VEGF showed cardinal features of the disease (Table 1).

### 2.1. VEGF Signaling in Stroke

Our review of anti-VEGF (inhibition) and pro-VEGF (stimulation) approaches to neurological diseases as summarized in Table 1 suggests that the clinically accepted VEGF inhibition therapy using bevacizumab and similar classes of monoclonal antibodies that inhibit all or certain isoforms of human VEGF is still lacking, including at its experimental stage, with the exception of ocular conditions such as age-related macular degeneration. There were preclinical studies on germinal matrix hemorrhage in prenatal rabbits and experimental hydrocephalus in young adult rats tested with angiogenesis inhibitors. In this relatively younger and wide spectrum of pre-middle age groups, anti-VEGF approaches have been proposed because the endogenous level of VEGF has not declined in the parenchyma adjacent to cerebral ventricles and in the cerebrospinal fluid (CSF). The neuroprotective effect of VEGF in cerebrovascular diseases is appreciated but the causal role of VEGF in the cerebral vasculature and ventricles has just begun to be revealed [8,12,16,18,19,20,22,24,28,29,30,31,32,33].

Physiologically, VEGF induces neuroprotective effect and neurogenesis. A pair of distinct elongated ridges on the floor of each lateral ventricle is the hippocampus. With aging, it has been demonstrated in studies using middle-aged or older rats that the level of VEGF is dramatically decreased in hippocampus in which learning and memory is centered. In this rat study, it has been shown that a subpopulation of astrocytes expressing two other growth factors and VEGF has declined in the hippocampus at post-middle age. The significance of this study is claimed that age-dependent reduction of neurogenesis is correlated with number of stem cells and neural progenitors in the dentate gyrus and that after middle age, the number of astrocytes who provide VEGF to maintain and protect neurons is less than that of the young ones while the total number of astrocytes has unaltered [34]. This study is further bolstered by the quantification of quiescent neural stem cells in the subgranular zone (SGZ) of hippocampus that it is not aging that reduces the neurogenic activity of hippocampal region. Rather, it is the microenvironment or the volume of vascular niche (capillaries) within the SGZ that exhibits an age-related decline [35].

**Figure 1:**
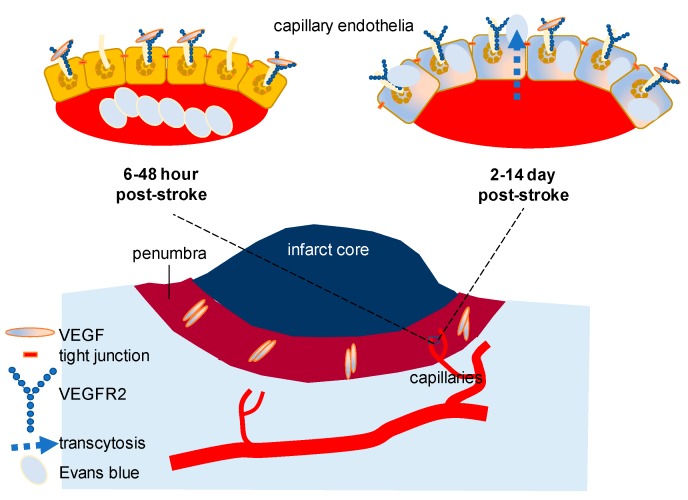
VEGF signaling in blood brain barrier (BBB) breakdown after stroke: after ischemic stroke, it has been demonstrated that BBB is disrupted and cerebral barrier is dysfunctional. Using young mouse model of diabetes, it has been recently demonstrated that VEGFR2 inhibition through SU5416 (semaxanib) improves BBB integrity of the brain regions adjacent to the infarct core after experimental ischemic stroke. Surprisingly, tight junction was intact while capillary endothelial transcytosis was markedly increased in the presence of edematous cellular morphology at ultrastructural level. Transcytosis of Evans blue dye was detected in the capillary endothelium in which loss of BBB integrity was identified at three days after stroke. Note that VEGF elevation reaches the peak 6–48 h after stroke with intermediate level of VEGFR2 (left) while total VEGFR2 elevation was at the highest level at 14 days after stroke. Maintenance of elevated VEGFR2 expression continues for a longer period than that of VEGF after stroke [32].

Pathologically, VEGF-induced endothelial permeability can alter BBB. For a small-molecule to cross the intact BBB, the expected molecular mass is less than a 400–500 Da [36]. Recent studies on stroke focusing on VEGF signaling have demonstrated that there is a dynamic alteration of VEGF and VEGF receptor (Figure 1). While a progressive decline of VEGF level is reported in the hippocampus with aging, the importance of VEGF receptor 2 (VEGFR2) expressed in the vascular endothelium has been highlighted in a more complicated pathological circumstance. Using a young adult mouse model of diabetes, it has been convincingly shown that inhibiting VEGFR2 improves functional outcomes and BBB disruption after stroke. Three major points of this study are that (1) the peak concentration of VEGF (the initial 6–48 h) and VEGFR2 (the initial 2–14 days) is detected in a different period after stroke thus inhibition approaches, if applied, should be adjusted based on the peak time of the highest VEGFR2, or in a delayed manner than previously thought; (2) BBB breakdown is due primarily to capillary endothelial transcytosis but not because of the tight junction alteration; and that (3) anti-VEGFR2 should be developed with a more personalized focus in a heterogeneous clinical population such as those patients with diabetes and stroke [32]. Although an inducible VEGFR2 knockout model was used as a validation for VEGFR2 inhibition through SU5416 (semaxanib) and there was a record of failure involving SU5416 in clinical trials in the past for other purposes, the result of this study was sufficient to be conclusive in testing hypothesis. This study has corrected our misunderstanding, if any, of BBB disruption in stroke and other cerebral circumstances with disintegrated barrier function: (1) Without loss or weakening of tight junction molecules in the endothelial cells, transcytosis of the otherwise impenetrable molecules (e.g., Evans blue, molecular weight 960.8 Da) is achievable to significantly increase the permeability of the cerebral capillaries in the presence of swelling or edema; (2) β-arrestin dependent endocytosis, which is known to be pivotal in vascular endothelial cadherin (VE-cadherin)-mediated endocytosis might not be the sole mechanism of VEGF-induced permeability in the cerebral capillaries [33], especially, during stroke with diabetes [32].

The finding reviewed in this section about stroke has focused on VEGF isoform A (VEGF-A) in vascular endothelial cells of the brain. A recent trend of an effort to find an alternative therapeutic approach of promoting neuroprotection other than VEGF isoform A is to focus on VEGF isoform B (VEGF-B) and cerebral pericytes [37]. In ischemic stroke, VEGF-B is claimed to play a role related with VEGFR1 signaling through regulation of the function of pericytes.

### 2.2. VEGF Signaling in Hydrocephalus: Lessons from Stroke

Hydrocephalus—a neurological condition sometimes associated with elevation of intracranial pressure—is a frequent neurosurgical target and characterized by excessive accumulation of fluid in the brain. The elevated amount of fluid accumulation measured by brain scan through computed tomography or magnetic resonance image has shown a robust correlation with cortical atrophy and related disorders. If untreated, hydrocephalus can cause prenatal mortality, neural cell death, excitotoxicity leading to an epileptogenic event and postnatal death. Hydrocephalus can occur regardless of the age and non-surgical treatments other than shunt surgery are rare and have been primarily reported in the postnatal and adult populations [38,39]. However, those in prenatal conditions or foetal-onset hydrocephalus have shown a limited surgical treatment managing flow of cerebrospinal fluid and that potential novel treatments have been limited until recently [38,40]. The timing of VEGF inhibition in early stage of ischemic or hemorrhagic stroke and that of pro-VEGF therapy in the stroke recovery phase following acute ischemia after SAH echoes the significance of VEGF signaling at a different pathogenic stage, age and concentration in hydrocephalus [12,16,41,42].

**Figure 2:**
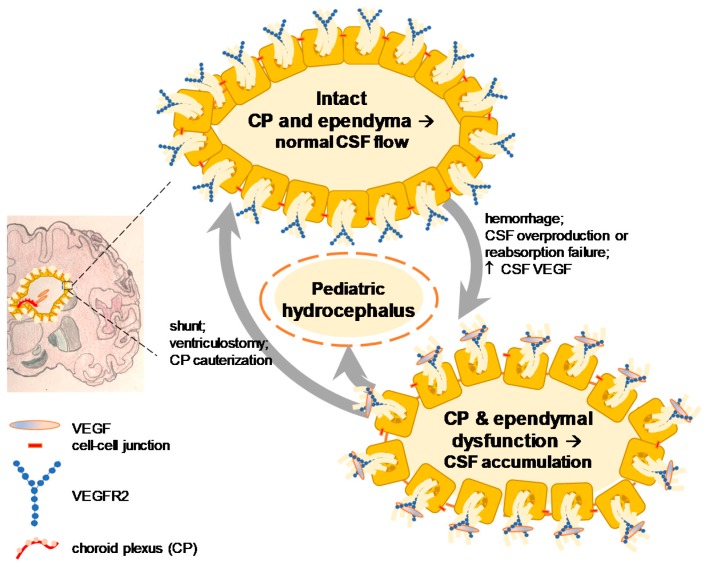
Role of VEGF signaling in post-hemorrhagic hydrocephalus from young individuals: pediatric patients with hydrocephalus have shown an elevated CSF VEGF up to 1 ng/mL [43] and that the infusion of the same dose VEGF into young adult rats results in experimental hydrocephalus. VEGF is considered pathologically causative and anti-VEGF therapy has been proposed [16]. CP, choroid plexus; CSF, cerebrospinal fluid.

Two independent groups have reported an apparently opposite but actually complementary result about the dual function of pathological and physiological VEGF signaling [12,21]. These studies measured the CSF VEGF of patients with hydrocephalus using the same method but the age and concentration of VEGF were mutually exclusive. The one who was investigating a neuroprotective effect of VEGF found that elderly patients (72.8 ± 3.2 years of age) with normal pressure hydrocephalus demonstrated a significant increase of VEGF (range: 10–70 pg/mL) in the CSF post-exercise as compared to no exercise controls (total sample size, 17 patients) [9]. On the other hand, another group studying a causative role of VEGF found that pediatric patients with hydrocephalus (median four years of age) exhibited a significant elevation of VEGF (range: 20 pg/mL to 1 ng/mL) in the CSF as compared to young control patients without hydrocephalus (median eight years of age) (total sample size, 66 patients) and showed that intraventricular infusion of VEGF at 1 ng/mL for 24 h resulted in experimental hydrocephalus six days later in rats [16] (Figure 2).

An interesting aspect of hydrocephalus might be its spectrum of similarity to ischemic or hemorrhagic stroke. Although stroke is distinguished into two subcategories when classifying cause-specific mortality in meta-analyses [44,45,46], the role of VEGF in stroke is not simple. Ischemic stroke can transform into hemorrhagic stroke. Likewise, hemorrhagic stroke can accompany an acute ischemic phase during a short moment right after intracranial hemorrhage. Ostrowski and Zhang have determined the pathological role of VEGF in a stroke model with hemorrhage: after induction of SAH in their rat model, they found that there is an acute ischemia due to rise in intracranial pressure and reduced cerebral perfusion with elevated VEGF in the cerebral cortex. In these rats with acute ischemia following hemorrhagic stroke, they showed hyperbaric oxygen treatment decreases early brain injury and reduces pathological VEGF [47]. This in fact has scientific foundation in timing of VEGF treatment in stroke. VEGF expression in early phase (1 h) after acute ischemic stroke is considered pathological and increases BBB leakage. In the late phase (48 h) after ischemic stroke, however, VEGF expression is considered neuroprotective, thus VEGF administration in this late phase reduces neurological deficits in the ischemic brain. The clinical significance of their study is that in the acute stage of stroke in which VEGF is pathological and breaks BBB integrity, VEGF inhibition is desired, while in the long term or late phase of stroke recovery stage, VEGF is protective against neural cell damage [17].

Because VEGF inhibition strategy is relatively new, active, and safe in terms of the side effect, an alternative approach for promoting VEGF signaling in stroke has been considered as an outstanding challenge. Briefly, three alternative methods of pro-VEGF therapy that might minimize the deleterious effect are suggested: (1) placental growth factor, a member of the VEGF subfamily in particular during embryogenesis through adeno-associated virus [48]; (2) VEGF-zinc finger protein through adenovirus and adeno-associated virus [49]; and (3) VEGF-mimetic peptide, QK [2,50]. In particular, it is noteworthy to distinguish the pros and cons of adenovirus over adeno-associated virus when delivering the target molecule or VEGF-zinc finger protein: the study described that adenovirus has high yield with short duration of transgene expression; adeno-associated virus is slower but allows sustained long-term expression of transgene [49].

Adeno-associated virus is adopted later and has been preferably utilized as a means of gene therapy in progressively developing pathological conditions because it has a different timing of transgene transduction as compared to the conventional methods [51]. Transduction of central nervous system using adeno-associated virus enabled a sustained expression of target gene for months with no adverse effect in humans. Efficacy and safety of adeno-associated virus gene therapy have demonstrated consistency in preclinical and clinical studies [52]. Phase-I clinical trials for patients under age of 70 years who received adeno-associated virus-glutamate decarboxylase 1 gene through intra-subthalamic nucleus injection and adeno-associated virus vector mediated delivery of aromatic l-amino acid decarboxylase gene through intra-putamen injection have shown to meet the goal in part [53,54]. Several clinical trials using adeno-associated virus are underway [55].

Tracing paradigms via axonal transport and viral vectors have been used to characterize form and functions of the specific neurotransmitter-releasing neurons. These techniques include wheat germ agglutinin along the axonal transport through microtubules. The development of genetically engineered tracing viruses, on the other hand, has extended the utility of this approach [56]. A recent method uses cell-specific promoter driven adeno-associated virus. The advantage of adeno-associated virus over other viral vectors is its low immunogenicity with a prolonged efficacy of transduction [57]. For example, the synapsin I protein, a member of the synapsin family that are neuronal phosphoproteins which associate with the cytoplasmic surface of synaptic vesicles has shown a neuron-specific transduction ability when combined with adeno-associated virus in vivo. One caveat, however, of this promoter is that viruses are often taken by unwanted types of neurons located at the site of injection, leading to non-specific labeling of unrelated neural pathways in the central nervous system. In contrast, a more specific expression of viral vectors has been suggested using neuron-subtype specific promoters [58]. The VEGF-zinc finger protein through adeno-associated virus shares the highly equivalent principle to this approach [58] and that it increases the specificity of transgene expression by using a zinc-finger protein transcription factor that targets the promoter region of VEGF [49]. These new techniques are currently applied to animal studies but as shown in clinical trials of adeno-associated virus for PD [53,54], pro-VEGF therapy using viral vectors as a clinically applicable optional treatment in cerebrovascular diseases including stroke and post-stroke hydrocephalus should be further developed.

### 2.3. An Alternative (EGFR/ErbB) Signaling in Stroke

If VEGF signaling were specific enough to treat cerebrovascular conditions, why would one consider other pathways? Oncologists had found that there is a strong correlation between elevated growth factors (VEGF and EGFR signaling) and a reduced survival time in patients with untreated cancer [59]. In an attempt to improve the diagnostic ability of predicting prognosis for lung cancer, specimens obtained from patients with previously untreated squamous cell lung carcinomas were assessed. In this immunohistochemistry of lung specimen clinicians found that VEGF and EGFR (or ErbB1 or Her1) are highly consistent biomarkers related with survival after diagnosis [59]. This report was further substantiated by the recurrence of cancer in patients treated with mono-therapy of targeting VEGF or EGFR alone [60]. This prompted an idea of multiple inhibition or double anti-angiogenic strategy [61]. One of the best-known anti-angiogenic therapies for cancer treatment had adopted VEGF inhibition (bevacizumab) in combination with EGFR tyrosine kinase inhibitor (Erlotinib) for patients with recurrent non-small-cell lung cancer [62]. Although anti-VEGF therapy, for instance with bevacizumab, does have side effects such as hypertension [63] and thereby careful treatment strategy is recommended [64], the success of clinical trials in cancer with double anti-angiogenic approaches has motivated researchers in cerebrovascular diseases to focus on both VEGF and EGFR signaling pathways.

Consistent with VEGF signaling (Table 1) [9,43,65,66], ErbB/EGFR signaling has been shown to exert beneficial as well as deleterious effects on the brain. In brain cancer research, VEGF has a major role in neovascularization of tumors, but other molecules such EGFR ligands are also involved and considered as important therapeutic targets. EGFR tyrosine kinase inhibitors (gefitinib and erlotinib) are clinically used in combination with VEGF or VEGFR2 inhibitor for glioblastoma [67]. ErbB network and related receptor signaling have been extensively reviewed primarily for cancer therapeutics and we recommend recent reviews on this pathway [67,68,69]. In non-neoplastic conditions, the beneficial effect of ErbB/EGFR ligands such as neuregulin has been shown in the brain with stroke. Based on the idea that elevated expression of neuregulin in neurons in the ischemic penumbra by focal stroke is neuroprotective, authors injected neuregulin to the rat model of stroke and found that neural cell apoptosis and pro-inflammatory response are prevented in the presence of a reduced infarct volume [70]. This study raises a question whether dual therapeutic approaches of providing neuregulin [70] and VEGFR2 inhibitor [68] at different time points can synergize neuroprotection and maintenance of BBB integrity after stroke.

In ischemic and hemorrhagic stroke, dual effects of several EGFR/ErbB ligands are noteworthy. Neuregulin-1, which is a ligand for ErbB4 receptor has shown a consistent neuroprotective effect in middle cerebral artery occlusion induced focal ischemia [71,72] as well as in ischemic phase (early brain injury) after SAH in rats [13]. The striatal infusion of transforming growth factor alpha (TGFα), which is a ligand for EGFR/ErbB1 has exhibited a similar neuroprotection in the rat model of ischemic stroke [72]. In a delayed treatment of HB-EGF infusion at one day after stroke induction, a reduced infarct size and neuroprotection against cell death have been reported in the ischemic rat study at the expense of compromised subventricular zone neurogenesis [73]. In human ischemic stroke patients, it has been demonstrated that systemic blood samples collected prior to hemorrhagic transformation have revealed a significant increase of amphiregulin, one of the EGFR/ErgB1 ligands. This report has concluded that amphiregulin may be a biomarker of hemorrhagic transformation in ischemic stroke [74].

In support of the interpretation of elevated amphiregulin in human blood prior to hemorrhagic transformation of ischemic stroke [74], the deleterious effect of ErbB/EGFR signaling has been demonstrated when HB-EGF is excessively expressed [12]. Using a transgenic mouse overexpressing ErbB1/EGFR ligand, HB-EGF, a group of investigators has shown that HB-EGF-mediated EGFR pathway is pathologically activated and stimulating VEGF signaling in the brain with SAH [12]. In an adjacent pathway, however, Yan and associates have described a protective role for ErbB4 in SAH based on its previous role on neuronal cell survival and death in neurodegenerative disorder and cerebral ischemia, respectively. They showed that ErbB4 signals along the Hippo pathway mediated by yes-associated protein1 (YAP), a transcription factor, and known to regulate cell proliferation in SAH. This study concludes that activation of the ErbB4-YAP signaling pathway is protective in a rat endovascular perforation SAH model [13] (Table 2).

### 2.4. Clinical Implication

Anti-VEGF therapy is approaching solving some clinical problems. Our understanding of what has hampered this has been the multifunctionality of the molecule: giving Avastin (bevacizumab) to some brain tumors may slow growth of some brain tumors, for example, but side effects of hemorrhage, edema, and hypertension might limit the use. The long-term resolution is to either make the drug more specific, or deliver it in a more specific way (such as by convection enhanced delivery). Similarly, using VEGF inhibitors to treat hydrocephalus is complicated by the same kinds of drug side effects, which may be worse than the condition treated.

However, the retinal diseases (macular degeneration and diabetic neovascularization) can be treated well, in part because the antibody can be delivered directly to the central nervous system (CNS) target by intravitreal injection, and the specific responses can be followed closely by ophthalmological examination [2,18,19]. The delivery of specific monoclonal antibodies directly to the retina (definitely part of the central nervous system) has revolutionized retinal medicine for these conditions. Given this, one can argue that bringing the treatment of other neurological conditions to this level would also change clinical care, even if it has not yet.

The causal role for VEGF has been questioned in stroke. Although the controversy has not been fully resolved, some of the latest studies have clarified the significance of VEGF level, at least, in ischemic stroke [75]. In this observation, a significant elevation of serum VEGF is reproduced in an internationally independent research group [75]. As such, consensus can be made as to the validation that elevated VEGF in ischemic stroke is “biomarker” for three subclasses of ischemic stroke, namely, atherothrombotic infarction, cardioembolic infarction, and lacunar infarction [75]. However, such a clinical report alone displaying VEGF as a biomarker (total human sample size, 171) is not sufficient to determine an appropriate therapeutic approach of pro-VEGF or anti-VEGF drugs. As described in this section, for clinical decision making of pro-VEGF therapy, however, a methodological advancement of delivering VEGF [30,51,52] or inducing endogenous VEGF-like trophic factors [49,58] are expected to achieve a more effective modulatory outcome of neuroprotection in the cerebrovascular disease.

## 3. VEGF Signaling in Age-Related Neurological Disorders

Effects of VEGF on the brain with cerebrovascular and neurodegenerative diseases have been previously reviewed by others [2,76,77,78]. One of the common features in these neurological conditions is BBB dysfunction as described in ALS, AD, and PD [2]. In particular, causal linkages of cardiovascular risk factors such as VEGF-mediated BBB dysfunction to AD has been claimed in the context of vascular health in age-related neuroprotection, resulting in “two-hit hypothesis” [36,77,78]. This vascular model hypothesized that vascular risk factors such as hypertension and diabetes in conjunction with genetic factors like ε4 allele of apolipoprotein E (APOE ε4) cause vascular damage first in the presence of BBB disruption and mild hypoperfusion or oligemia (hit 1). Then, insufficient clearance and excessive accumulation of Amyloid beta, Aβ, (hit2) lead to neurodegeneration and dementia. In these apparently irreversible degenerative circumstances, how VEGF mediates BBB dysfunction is in question [2]. Because VEGF is originally found as vascular permeability factor, there is no doubt that VEGF alters BBB through induction of endothelial hyperpermeability. While VEGF-induced endothelial permeability is clear [33,38,79], a direct or indirect involvement of VEGF in breakdown of junctional molecules in BBB or transport across intact BBB might have three possibilities: (1) tight junctions; (2) adherens junctions; and (3) transcytosis [32,79]. Usually, BBB dysfunction is thought to accompany breakdown of tight junctions [36,77,78]. However, a recent study raises a new possibility that penetrance of large molecules (>400 Da) across intact BBB is reported (Evans blue dye, 960.8 Da) in the presence of intact tight junctions, elevated VEGF, and elevated VEGFR2 [32]. Because Evans blue extravasation in the vicinity of penumbra was evident, this might be dysfunctional BBB but intact tight junctions [32]. Even if BBB dysfunction is a feature of neurodegenerative diseases such as ALS, AD, and PD, insufficient neuroprotection is a fundamental problem in these conditions with increasing age. In ALS, low VEGF is reported with BBB dysfunction. Thus, therapeutic use of VEGF is validated using a drug including VEGF-A_165_ in the recent clinical trial (NCT02269436) for patients with ALS [2]. Together, specific molecules of ApoE ε4 in AD and VEGF (VEGF^δ/δ^ mice) in ALS support the idea that age-related decline in vascular healthiness increases the risk of AD and that of ALS, respectively [2,36,77,78]. As such, the causal relationship between VEGF and these age-related neurological conditions is clearer in ALS and in AD than PD. For PD, then, are there specific molecules like ApoE or VEGF or others directly linked to common cardiovascular risks such as hypertension, atherosclerosis, and diabetes? To answer this question, we reviewed both VEGF and vascular risk factors in relations to the disease below.

### 3.1. VEGF Levels in AD

VEGF levels in AD have been actively studied in patients but the results are less consistent. The CSF VEGF has been demonstrated to increase in patients with AD in a relatively small number of samples [80]. In a more recent independent study with 92 controls and 69 AD patients, CSF VEGF levels have been significantly decreased in AD (14.49 ± 1.90 pg/mL) as compared to the control (15.30 ± 1.87 pg/mL) group [81]. Although a significant difference is obtained during statistical analysis, a criticism can arise because the magnitude of CSF VEGF in both groups is similar to the assay detection limit (the lowest at 15.6 pg/mL) found in other studies measuring human CSF, which adopted the same method [12,43]. When considering a particular VEGF variant of the C(-2578)A genotype, the level of VEGF in the serum is significantly increased in AD patients carrying the AA genotype compared with AD patients carrying the CA or CC genotypes [82,83]. In other studies, reduced serum VEGF is reported in patients with AD [84,85].

Using a stereologically implanted encapsulated VEGF-secreting cells, it has been shown that cognitive impairment of APP/Ps1 mouse model of AD has been improved when animals are treated with VEGF, suggesting that VEGF is neuroprotective [25,86]. In the same mouse model of AD, VEGF loaded nanosphere has been shown to enhance hippocampal neurogenesis [26]. Based on the premise that earliest cerebral hypoperfusion is associated with AD in the medial parietal cortex, the elevated VEGF has been detected in this region of post-mortem tissues from 70 patients with AD as compared to 37 control specimens [27]. As to one of the noteworthy VEGF isoforms, there is one recent study reporting that VEGF-B might be a novel biomarker for early detection of clinical AD, although more study is necessary [87].

### 3.2. VEGF Levels in PD

As a long-term inhibition of VEGF signaling shown in some cases of cancer treatments leads to vascular stiffening, VEGF is also involved in extra-vascular pathological conditions such as PD. VEGF levels are known to decrease with increasing age in normal healthy brains [2]. In animal models and human clinical specimens of PD, however, there are conflicting reports of VEGF expression levels in the region along the nigrostriatal neural connection [88,89,90]. PD, an age-related neurodegenerative disorder is characterized by tremor, rigidity and motor dysfunction, caused by degeneration of dopaminergic neurons in the brain, particularly, substantia nigra pars compacta (SNc) and highlighted, pathologically, by the formation of fibrillar aggregates or Lewy bodies in surviving neurons of various regions [91,92]. Despite limitations, neurotransmitter replacement with levodopa (l-DOPA) is the mainstay of treatments [93]. Clinical manifestation and existing models have suggested accumulation of α synuclein (α-syn), exposure to 1-methyl-4-phenyl-1,2,3,6-tetrahydropyridine (MPTP), and mutations in PD associated genes (PARK) as known cause, but 95% of cases remains idiopathic [94]. Sporadic or idiopathic PD is a slowly progressive disease with a monogenic or multifactorial pathogenic mechanism that develops over decades before motor symptoms appear [91,94].

A recent rat study reported that nigral vessel density and VEGF mRNA have decreased with increasing age and that such an age-dependent loss of VEGF is reversed by physical exercise [88]. There is an early report attempting to elucidate the relationship between dopamine and angiogenesis showing that dopamine, which is significantly decreased in substantia nigra pars compacta of patients with PD, inhibits VEGF-induced angiogenesis by inducing endocytosis of VEGFR2, thereby, blocking the binding of VEGF [95]. Therapeutic use of VEGF-A has been tested through transplantation of human umbilical cord mesenchymal stem cells pre-modified by adenovirus-mediated VEGF gene transfer in rotenone-lesioned hemiparkinsonian rats [96]. In addition to beneficial effects of VEGF-A [97,98], an endogenous level of VEGF-B has been also shown to increase and that exogenous VEGF-B has also shown a neuroprotective effect in a culture model of PD [99].

Unlike these reports, a rotenone-induced in vivo model of PD has revealed the opposite trend. VEGF expression in the substantia nigra is decreased when Parkinsonian phenotype of reduced striatal dopamine and reduced nigral tyrosine hydroxylase (TH) are experimentally induced and that l-DOPA treatment in combination with other drugs delayed the decrease of striatal VEGF levels [89]. In support of this animal study, there is a recent report claiming that VEGF protein is upregulated in the substantia nigra but not in the striatum of patients with PD when measured using enzyme linked immunosorbent assay [90]. In the CSF, VEGF and other angiogenic markers have been shown to elevate in patients with PD [28]. Using intranasal delivery of desferrioxamine, an iron chelator widely used in clinical settings, it has been shown that Parkinsonian symptoms of MPTP intoxication induced nigral degeneration have been improved in the presence of elevated VEGF protein levels in the nigrostriatal tissue [29]. VEGF levels in the serum of patients with PD have been assessed but the studies failed to associate serum VEGF with idiopathic PD [100,101].

Given the possibilities of monogenic, multigenic, and blending theory of genetic and environmental causal factors, BBB dysfunction as causative mechanism has been noted in PD [102]. As such, compromised BBB integrity is thought to contribute to the pathogenesis of PD by reactive gliosis, which in turn results in elevated secretion of VEGF and proinflammatory cytokines by activation of astrocytes and microglia [2]. Low and high VEGF are shown to lead to an opposite effect on dopaminergic neurons in a toxin-induced PD model. Consistent with recent reports [2], low dose VEGF exerts a neuroprotective effect while high dose VEGF results in edema, hyperpermeability, and deleterious effect on dopaminergic neurons [103]. As reported recently in patients with fibromyalgia syndrome, arterial stiffness and VEGF are inversely correlated [104]. In other words, insufficient VEGF increases arterial stiffness whereas excessive VEGF increases permeability, thereby, decreasing vascular stiffness. In this context, it is interesting to note that patients with PD show elevated CSF VEGF and reduced arterial stiffness [2,28,105,106,107,108].

Similar to the recent trend in an alternative isoform study of VEGF [37], exogenous VEGF-B has been shown to exert a neuroprotective effect in PD models [109].

### 3.3. VEGF and Other Vascular Risk

**Figure 3:**
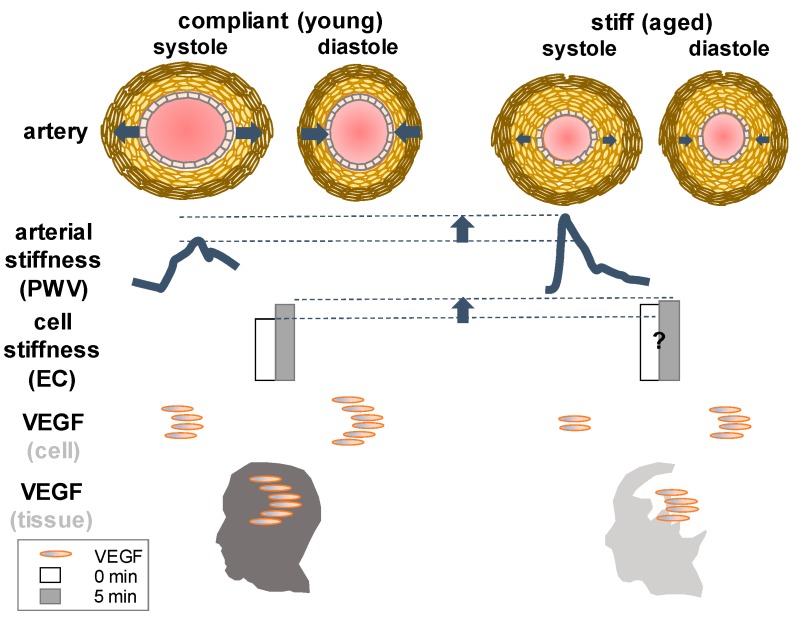
An increase of VEGF contributes to an increase of endothelial cell stiffness at an instantaneous time period of minutes: left—a temporal increase of cell stiffness is exhibited to be a response to external force or shear stress in aortic endothelial cells [111]. Cellular stiffness quantified by the number of focal adhesions in endothelial cells (ECs) before (zero minutes) and after applying force (five minutes) is shown in simplified bars [111]. An increase of VEGF is reported to contribute to an increase of extracellular matrix stiffness of tumor endothelial cells in vitro [112]; right—vascular stiffness measured by pulse wave velocity (PWV) has been known to significantly increase in normal healthy individuals over aging [113], while VEGF levels, for example, in the brain decrease with increasing age at tissue level [2]. Illustration is modified from [114,115,116]. A question mark denotes the interpretation by present authors based on previous reports about changes in vascular stiffness at cellular level over a long period of aging [113,117] that needs a further clarification.

VEGF is known to modulate vascular contractility and blood pressure. Several recent studies have suggested that vascular stiffness (Figure 3) may contribute to hypertension seen in patients treated with VEGF inhibitor (bevacizumab) or VEGF kinase inhibitors [110].

Mechanisms of hypertension associated with sorafenib, for example, an inhibitor of Raf kinase and VEGF receptor 2 have been implicated as an increase of vascular stiffness during the period of anti-cancer drug treatment accompanying the side effect of elevated systolic blood pressure [118]. With increasing age, vascular stiffness in large arteries like aorta significantly increases while VEGF levels decline in the brain. The relationship between VEGF and compliance or stiffness is not as straightforward as the relationship between VEGF and permeability or a pressure-volume curve of the blood vessel, which has been described in the lexicon of cerebral pulse wave or intracranial pulsatility that is finely tuned with arterial blood pressure waveform in the pulsating brain [119]. It will be simpler if altered permeability results in consistent alterations of stiffness or compliance or vice versa. For instance, if all other conditions are homogenous (chemical composition) and isometric (orientation), permeable vessels (elevated VEGF) are more porous than impermeable ones thereby less stiff. However, in cancer and age-related pathological state, such an assumption (homogeneous chemical composition and isotropic properties) is inapplicable, leading to inconsistency. Over a long period of aging, care should be taken to relate VEGF with tissue stiffness even in normal healthy individuals (Figure 3). For more details about arterial blood pressure waveform and intracranial pressure along with VEGF we recommend previous reviews [2,119,120].

Stiffening of arteries, either in aorta or small arteries and arterioles, has been associated with hypertension. It is an independent cardiovascular risk factor reversibly forming a transition between health and disease. Aortic stiffness is known to accurately predict the development of cardiovascular disease [113]. While there is a little gap between the blood pressure measurement between small animals (invasive arterial cannulation) and humans (non-invasive), the way of measuring arterial stiffness in vivo is highly consistent between the species. The elevated magnitude of arterial stiffness measured by pulse wave velocity using Doppler ultrasound has shown a strong correlation with cause-specific mortality in the aged populations [121]. In a man older than 70 years of age, an increase in pulse wave velocity is directly associated with death due to cardiovascular disease. Arterial stiffening develops due to changes in structural and functional properties of the blood vessels. In aortic media, for example, an increase of smooth muscle cell, a primary source of VEGF secretion in the arteries is shown to alter stiffness. If untreated, arterial stiffening can be causative to pathogenesis of cardiovascular diseases [110,112,122].

The recent studies suggest that forced long-term inhibition of VEGF signaling through VEGF kinase inhibitors gives rise to a deleterious effect of vascular stiffening that may evoke hypertension (Table 3).

In non-neoplastic conditions, one of the clearest examples for the relationship between VEGF and tissue stiffness would be hepatic fibrosis or cirrhosis in liver. It is well documented that magnetic resonance elastography similar to Doppler ultrasound are actively utilized in relating wave propagation along the tissue (liver) of interest to the degree of tissue compliance or elasticity [123,124,125]. Both techniques are known to accurately predict or diagnose the tissue elasticity of living patients. In this circumstance with fibrotic liver, VEGF contribution to the formation of perivascular fibrosis has been shown to be complex but consistent with other tissues such as brains that inhibiting VEGF signaling with a small molecule receptor tyrosine kinase inhibitor SU11248 significantly reduces hepatic vascular density. This suggests that therapies against angiogenesis through VEGF signaling might be effective [61]. The magnetic resonance elastography has been applied recently to the brain, although it is yet to be conclusive in hydrocephalus [126].

### 3.4. Vascular Risk Factors in Neurological Disorders

We have summarized diverse roles of VEGF in several neurological conditions (Table 1). As we reviewed the potential dual impact of VEGF and found that the causal linkage of VEGF in PD is less clear than that of other age-related neurological diseases such as ALS (e.g., VEGF^d/d^ mice [2]) and AD (e.g., two-hit hypothesis [36,77,78]), we asked what the relationship would be between vascular stiffness and neurological disorders. An independent and highly reproducible cardiovascular risk factor similar to vascular stiffness in the clinic and animal studies alike has been sought to more thoroughly understand neurovascular interactions in the brain under age-related neurodegenerative conditions.

The literature indicates that vascular risk factors such as vascular stiffness, intraocular pressure (IOP), cerebral pulsatility, intracranial pressure, and smoking showed a consistent relationship with the disease.

As expected, aging increases vascular stiffness in normal healthy individuals [127]. In the neurological impairment of eye such as glaucoma, the ocular disease and IOP showed a proportionate relationship [128]. In an early phase after infarct or stroke, intracranial hypertension due to ischemia-induced vascular leakage and edema, blood pressure rise is a critical indicator of an adverse event hampering stroke recovery [129]. During collateral formation and reparative angiogenesis, however, a response of vascular factors or VEGF is varied and different than the early phase of stroke [2]. In pediatric or young adult ages, intracranial pressure and cerebral pulsatility are proportionately associated with hydrocephalus [119,120]. In normal pressure hydrocephalus of aged patients, no significant association is detected between the factor and the disease [130].

In ALS, diurnal variation of blood pressure is reported but we failed to discern a clear relationship between a cardiovascular risk factor and the disease [2,23,131,132]. In AD, an increase of vascular stiffness is significantly correlated with cognitive decline [133]. In PD, however, there were many reports highlighting the inverse relationship between the vascular risk factor and the disease. Of surveyed risk factors, cigarette smoking has been repeatedly pointed out as an intriguing indicator exhibiting an unusual association with the disease [105,106,134,135,136,137,138] (Table 4).

The literature survey indicates that an inverse relationship between smoking and PD could be explained alternatively: (1) patients with PD tend not to smoke or tend more likely to quit smoking earlier than others because they are lacking nicotine receptors, thereby, nicotine-induced reward system (dopamine) does not work in those destined to PD; (2) In other words, ease of smoking cessation is an aspect of premanifest PD similar to olfactory dysfunction; (3) nicotine, is indeed neuroprotective against the loss of dopaminergic neurons, which fairly explains why there is a consistent trend of more non-smokers in patients with PD than control groups in multiple cohort studies. Nicotine is a potent parasympathomimetic stimulant found in the plants. It is a highly addictive chemical alkaloid and previously used as an insecticide, implying that this is a toxin at high enough concentrations. In patients with PD, transdermal nicotine treatment has shown an improvement of motor score during a pilot clinical trial [139]. In an intact porcine common carotid artery perfusion culture model, it has been demonstrated that nicotine causes a significant elevation of VEGF, particularly in endothelial cells [140]. Given the detrimental cardiovascular consequence of smoking, the vascular aspects of PD via ‘stiffness measure’ along with VEGF assay warrant a more thorough study [135,136,137,138,139,140,141,142,143].

## 4. Materials and Methods

The literature search was conducted using PubMed database. We searched ligands of VEGF or VEGF-A because it is the original growth factor found as vascular permeability factor. We used the keywords: “VEGF and stroke”; “VEGF and hydrocephalus”; “VEGF and ocular disease”; “VEGF and age-related macular degeneration”; “VEGF and glaucoma”; “VEGF and diabetic retinopathy”; “VEGF and amyotrophic lateral sclerosis”; “VEGF and Alzheimer’s disease”; and “VEGF and Parkinson’s disease”. We then iterated the search using “VEGF-B”.

To include prior studies of EGFR, we searched ligands of ErbB1/EGFR, ErbB2, ErbB3, and ErbB4. We found that there are many ErbB/EGFR family ligands specifically binding ErbB1 and ErbB4. Then, we searched literatures studying ErbB1 ligands or ErbB4 ligands in association with stroke. On this basis, we made a table and briefly described in the text. The ligands we found actively studied in recent are neuregulin (ErbB4 ligand), amphiregulin (ErbB1 ligand), TGFα (ErbB1 ligand) and HB-EGF (ErbB1 and ErbB4 ligand) in stroke research field.

## 5. Conclusions

The review of the literature suggests that VEGF signaling in the cerebrovascular disease, especially ischemic stroke and post-hemorrhagic hydrocephalus, highlights the importance of concentration, timing, and alternate route of modulating VEGF signaling pathway. In age-related neurological conditions, cerebral barrier dysfunction is noted with elevated VEGF. An intriguing inverse relationship between vascular risk factors and some age-related neurological conditions warrants a further study. VEGF is approaching solving some clinical problems but there is an issue of the multifunctionality or an adverse effect. The long-term resolution is to either make the drug more specific, or deliver it in a more specific way such as by convection enhanced delivery. Similarly, using VEGF inhibitors to treat hydrocephalus is complicated by the same kinds of drug side effects.

Using a similar anti-VEGF drug, however, the retinal diseases have been treated well, in part because the antibody can be delivered directly to the CNS target by intravitreal injection, and the specific responses can be followed closely by ophthalmological examination. The delivery of specific monoclonal antibodies directly to the retina has revolutionized retinal medicine for these conditions. Given this, bringing the treatment of other neurological conditions to this level would also change clinical care. Together, molecular targets distinguishing two distinct pathways of VEGF signaling may provide novel therapies for the treatment of age-related cerebrovascular and neurological diseases and for maintaining lower mortality due to these conditions.

## Figures and Tables

**Table 1:** Vascular endothelial growth factor (VEGF) signaling in neurological disorders.

Disease	VEGF Level (Where)	Concurrent Cerebral Events	Inhibit VEGF?	Promote VEGF?	Reference
Ocular disease	↑ VEGF (ocular capillaries)	leaky microvasculature	yes		[18,19] ^m^
Stroke (early phase)	↓ VEGF; ↑ VEGF (cerebral cortex)	leaky vasculature	yes		[17] ^r^
Stroke (collateral forming phase)	↓ VEGF (cerebral cortex)			yes	[17] ^r^
	↑ VEGF (cerebral cortex)	reparative angiogenesis		yes	[20] ^m^
Hydrocephalus (young)	↑ VEGF (CSF)	ventriculomegaly and hemorrhage	yes		[12] ^m^, [16] ^h^, [8] ^rabb,h,^*
Hydrocephalus (aged brains)	↑ VEGF (CSF)	a slight increase in intracranial pressure (NPH)		yes	[9,21] ^h^
ALS	↓ VEGF (cerebral cortex)	insufficient neuroprotection; motor neuron degeneration; BBB dysfunction		yes	[22,23] ^h^
AD	↑ VEGF (cerebral cortex)	cerebral hypoperfusion; neural & BBB dysfunction and loss		yes	[24,25,26] ^m^, [27] ^r^
PD	↑ VEGF (CSF)	leaky vasculature; white matter lesion		yes	[28] ^h^, [29] ^m^

c.f. ^m^, mouse; ^r^, rat; ^rabb^, rabbit; ^h^, human; ALS, amyotrophic lateral sclerosis; AD, Alzheimer’s disease; PD, Parkinson’s disease; CSF, cerebrospinal fluid; * VEGF level measured in prenatal germinal matrix.

**Table 2:** Erythroblastic leukemia viral oncogene (ErbB)/EGFR signaling in stroke.

Disease	Ligand Level (Where)	Concurrent Cerebral Events	Inhibit ErbB?	Promote ErbB?	Reference
Stroke (ischemic)	↑ neuregulin (cerebral cortex)	neuroprotection		Yes (ErbB4)	[71] ^r^
	↑ neuregulin (cerebral cortex)	reduced apoptosis		Yes (ErbB4)	[70] ^m^
	↑ TGFα (striatal infunsion)	neuroprotection; neural migration		Yes (ErbB1)	[72] ^r^
	↑ HB-EGF (icv infusion)	neuroprotection; reduced infarct size		Yes (ErbB1; ErbB4)	[73] ^r^
	↑ amphiregulin (systemic blood)	hemorrhagic transformation	Yes (ErbB1)		[74] ^h^
Stroke (hemorrhagic)	↑ neuregulin (cerebral cortex)	neuroprotection after SAH		Yes (ErbB4)	[13] ^r^

c.f. ^m^, mouse; ^r^, rat; ^h^, human; icv, intracerebroventricular; SAH, subarachnoid hemorrhage.

**Table 3:** VEGF signaling in vascular stiffening.

Condition	VEGF Level (Contributor)	Symptoms	Reference
Pulmonary artery stiffening	↑ VEGF (elevated pulsatility)	Reduced compliance Pulmonary hypertension	[122]
Solid tumor progression	↑ VEGF (matrix cross-linking)	Increased matrix stiffness Increased matrix metalloproteinase activity	[112]
Anti-cancer treatment	↓ VEGF (VEGF kinase inhibition)	Increased hypertension	[110]

**Table 4:** A relationship between vascular risk factors and neurological disorders.

Condition	Vascular Factor	Interrelationship	Reference
Aging	vascular stiffness (pulse wave velocity, PWV)	Proportionate ↑ aging → ↑ PWV: yes ↑ PWV → ↑ aging: likely	[127]
Ocular disease	Intraocular pressure, IOP	Proportionate ↑ glaucoma → ↑ IOP: yes ↑ IOP → ↑ glaucoma: likely	[128]
Stroke (early phase)	blood pressure (hypertension)	Proportionate ↑ blood pressure → ↑ stroke: yes ↑ stroke → ↑ blood pressure: likely	[129]
Stroke (collateral forming phase)	blood pressure	Varied	[2]
Hydrocephalus (young)	cerebral pulsatility (↑ intracranial pressure)	Proportionate ↑ hydrocephalus → ↑ pulsatility: likely ↑ pulsatility → ↑ hydrocephalus: likely	[119,120]
Hydrocephalus (aged brain)	intracranial pressure	No significant association (normal pressure hydrocephalus)	[130]
ALS	blood pressure (diurnal variation)	varied with chronic ischemia, lack of neuroprotection, and autonomic failure	[2,23,131,132]
AD	vascular stiffness (PWV)	Proportionate ↑ AD → ↑ PWV: yes ↑ PWV → ↑ AD: not always but likely	[133]
PD	vascular stiffness (PWV)	Inverse ↑ PD → ↓ PWV: most likely ↓ PWV → ↑ PD: not always but likely	[105,106]
	Smoking	Inverse ↑ smoking → ↓ PD: yes ↓ smoking → ↑ PD: likely ↑ PD → ↓ smoking: yes (easier to quit) ↓ PD → ↑ smoking: not always but likely	[134,135,136,137,138]
